# Overcoming current challenges to T-cell receptor therapy *via* metabolic targeting to increase antitumor efficacy, durability, and tolerability

**DOI:** 10.3389/fimmu.2022.1056622

**Published:** 2022-11-21

**Authors:** Wendy Mao

**Affiliations:** Cell Biology, BioNTech Societas Europaea (SE), Gaithersburg, MD, United States

**Keywords:** metabolism, T-cell receptor therapy, adoptive cell therapy, tumor microenvironment, cancer immunology and immunotherapy, T cell fitness

## Abstract

The antitumor potential of personalized immunotherapy, including adoptive T-cell therapy, has been shown in both preclinical and clinical studies. Combining cell therapy with targeted metabolic interventions can further enhance therapeutic outcomes in terms of magnitude and durability. The ability of a T cell receptor to recognize peptides derived from tumor neoantigens allows for a robust yet specific response against cancer cells while sparing healthy tissue. However, there exist challenges to adoptive T cell therapy such as a suppressive tumor milieu, the fitness and survival of transferred cells, and tumor escape, all of which can be targeted to further enhance the antitumor potential of T cell receptor-engineered T cell (TCR-T) therapy. Here, we explore current strategies involving metabolic reprogramming of both the tumor microenvironment and the cell product, which can lead to increased T cell proliferation, survival, and anti-tumor cytotoxicity. In addition, we highlight potential metabolic pathways and targets which can be leveraged to improve engraftment of transferred cells and obviate the need for lymphodepletion, while minimizing off-target effects. Metabolic signaling is delicately balanced, and we demonstrate the need for thoughtful and precise interventions that are tailored for the unique characteristics of each tumor. Through improved understanding of the interplay between immunometabolism, tumor resistance, and T cell signaling, we can improve current treatment regimens and open the door to potential synergistic combinations.

## Introduction

**Figure 1:**
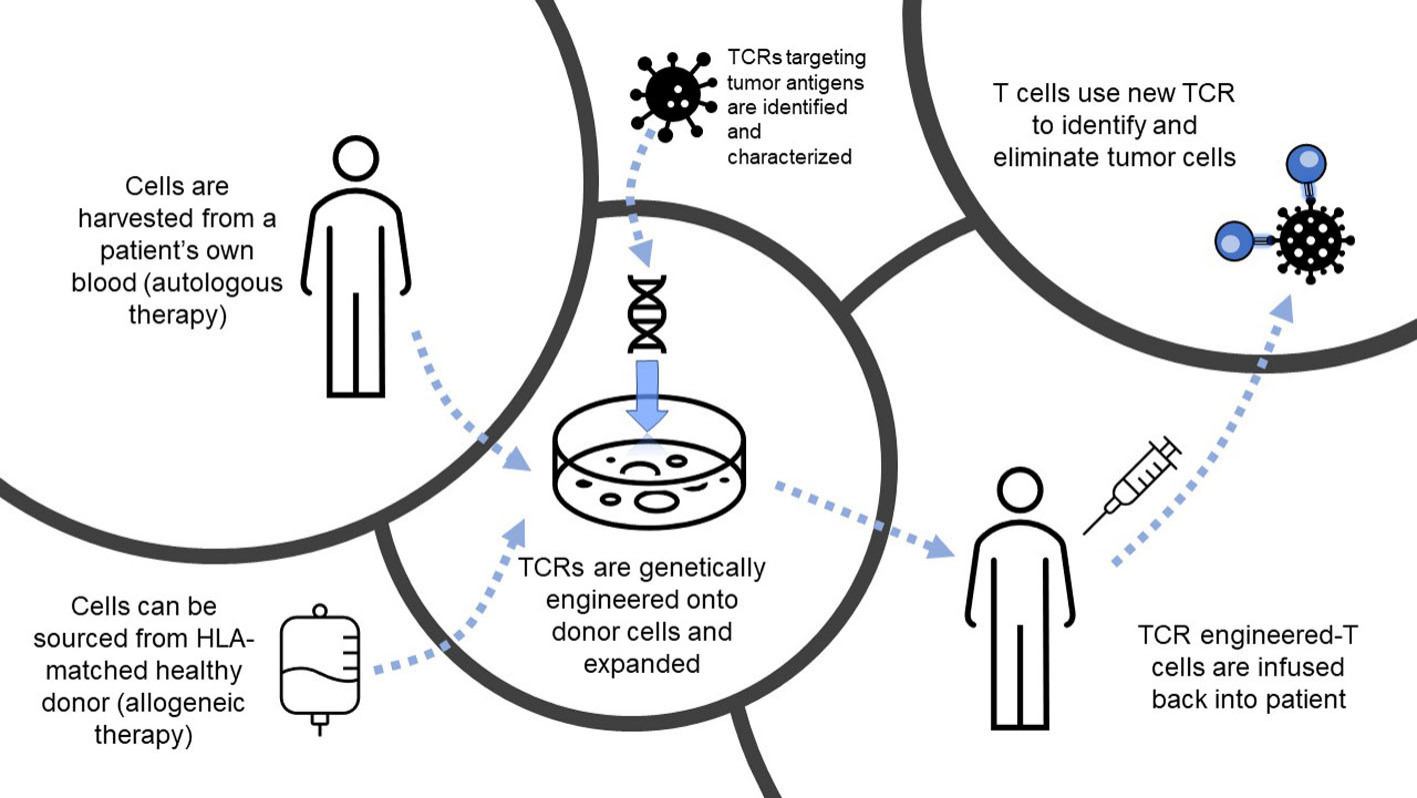
Overview of T cell receptor-engineered T cell (TCR-T) therapy. Depiction of the process of TCR-T, from autologous or allogeneic cell sourcing to engineering and infusion to target tumor cells. HLA, human leukocyte antigen; TCR, T cell receptor.

**Figure 2:**
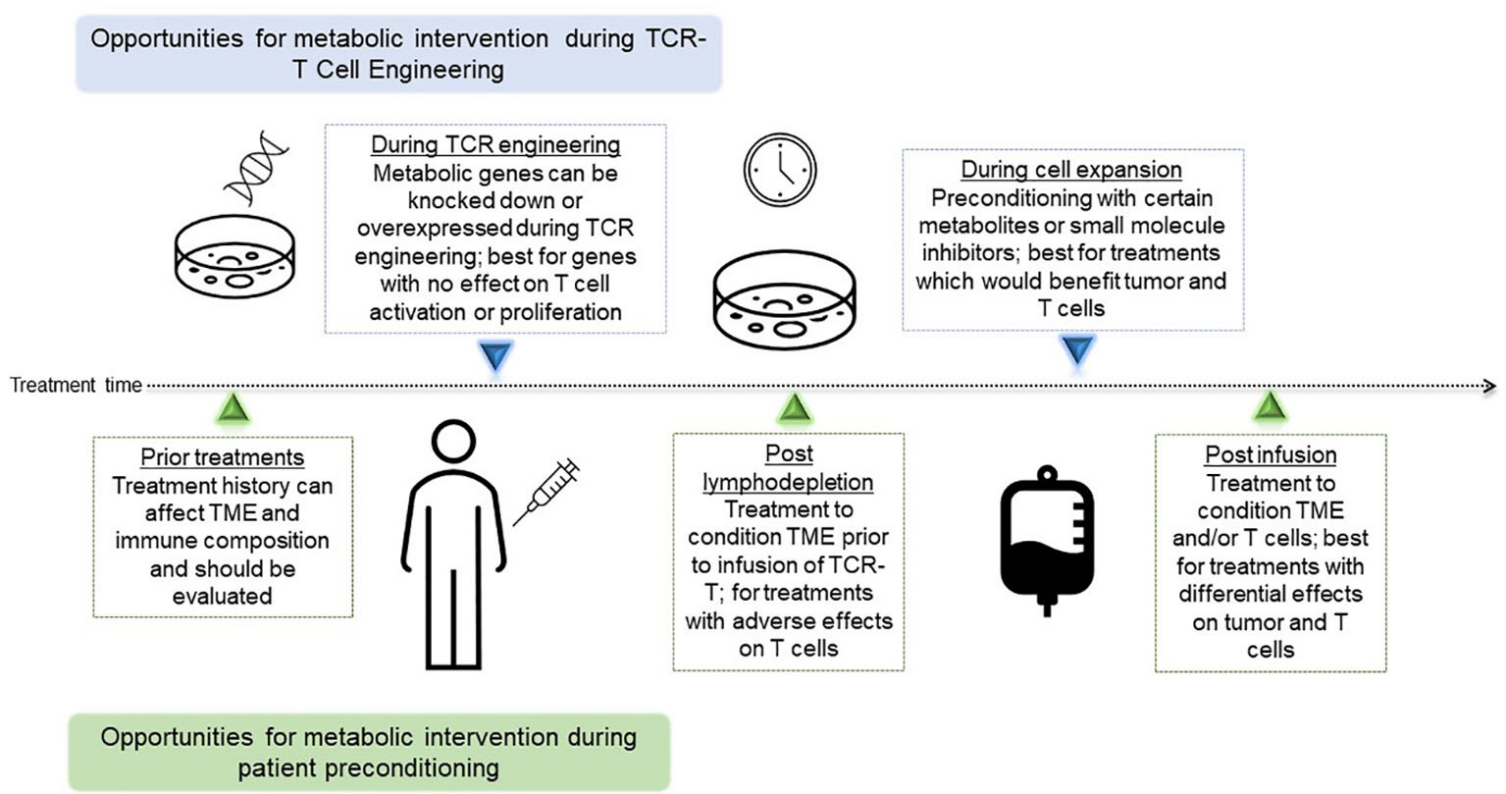
Timing of Opportunities for metabolic interventions during TCR-T. Depiction of metabolic interventions that can be deployed as part of the cell engineering and expansion process, or part of patient preconditioning and treatment. TCR-T, T cell receptor-engineered T cell; TCR, T cell receptor; TME, tumor microenvironment.

Success of immunotherapy directed against cancer is predicated on the fact that tumor cells can be specifically targeted by various populations of immune cells, minimizing damage to healthy tissue while exclusively eliminating cancerous growth. Adoptive cellular therapy (ACT) for solid tumors has gained increased attention in recent years following its initial success in metastatic melanoma (1) and more recent promising results in lung (2) and pancreatic (3) cancers, demonstrating the potential for adoptively transferred tumor-antigen specific T cells to mediate an antitumor response. In this review, we will focus on TCR-T, which has already shown promising clinical efficacy in solid tumors (3–5). In this modality of ACT, T cell receptors (TCRs) targeting tumor antigens presented in the context of major histocompatibility (MHC) molecules are engineered onto isolated T cells and then infused into a patient, which will then mediate tumor regression (6) (**Figure 1**). Despite its successes, the field of ACT has faced many challenges, from inadequate immune infiltrate, a suppressive tumor microenvironment (TME), tumor heterogeneity and evolution, and exhausted effector cells, among others. Despite preclinical promise, many TCR-T therapies provide only limited benefit in clinic (7, 8), while in other contexts, tumors relapse after initial remission following treatment (9), suggesting that immune suppression and tumor evolution can dampen success of these therapies. Metabolic interventions hold promise for enhancing TCR-T survival, persistence, trafficking, and cytotoxicity against nutrient-devouring tumor cells. The TCR-T treatment process offers many opportunities for metabolic intervention, with the ability to precondition both T cells and the tumor separately prior to infusion to achieve the most benefit. These include treatment modalities that can be applied during engineering to attain better cell phenotypes, as well as systemic treatments pre-and post-infusion to condition the TME, each of which will be evaluated in subsequent sections, and are summarized in **Figure 2**. In this review, we will discuss methodologies of targeting metabolic pathways to not only increase the antitumor efficacy and durability of TCR-T, but also improve engineering and clinical protocols to further extend the capabilities of ACT.

## Strategies for targeting tumor metabolism to improve T cell fitness

**Figure 3:**
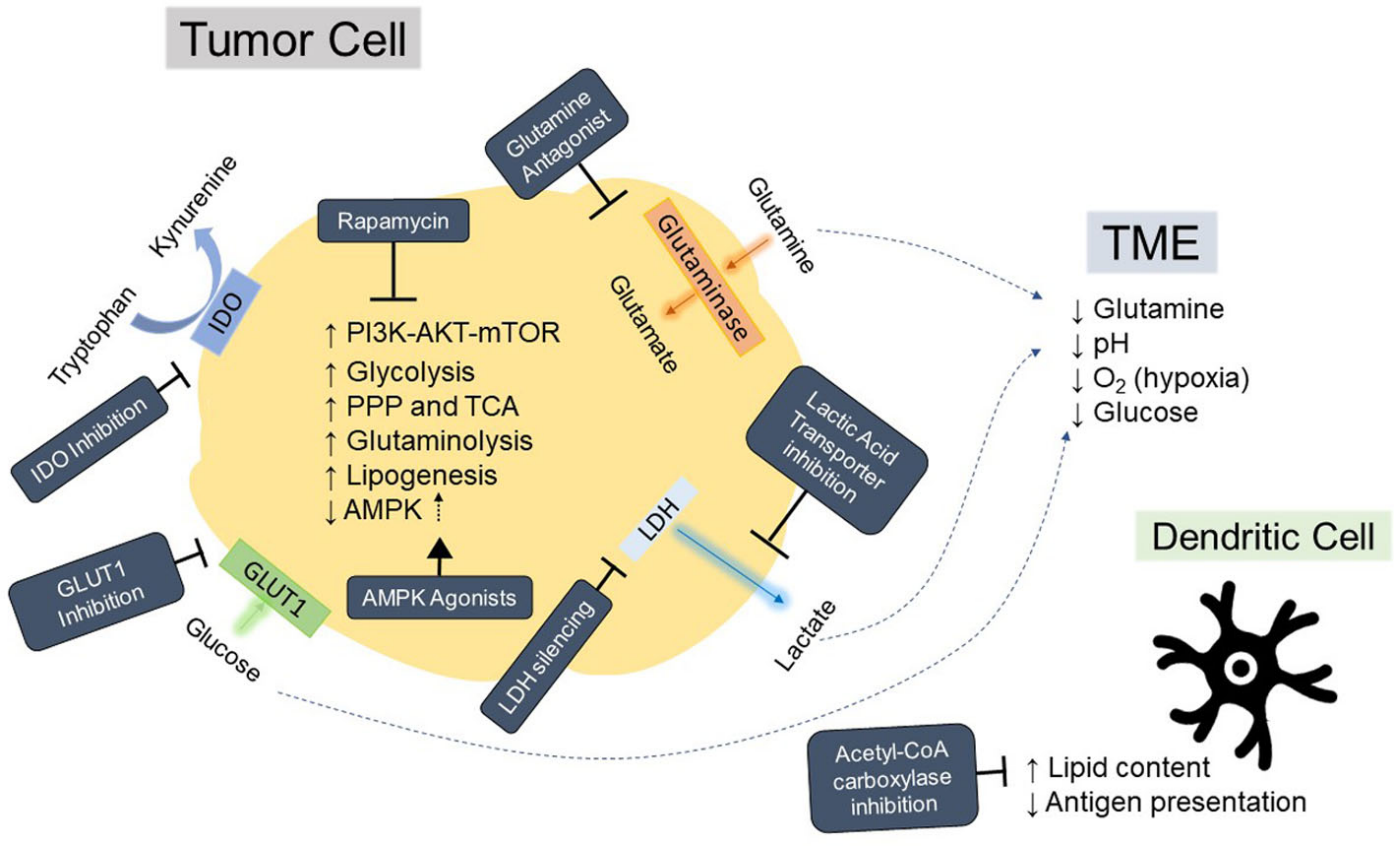
Hallmarks of tumor metabolism fueling immunosuppressive tumor microenvironment and strategies for modulation. Depiction of aspects of tumor metabolism that contribute to acidification, nutrient deprivation, and immunosuppression in the tumor microenvironment (TME) as well as general mechanisms for inhibiting these pathways. Strategies for inhibition or reversal of tumor phenotypes are in dark blue boxes with white text. AMPK, AMP-activated protein kinase; PI3K, Phosphoinositide 3 kinase; AKT, protein kinase B; mTOR, mammalian target of rapamycin; IDO, Indoleamine 2, 3-dioxygenase; LDH, lactate dehydrogenase; PPP, pentose phosphate pathway; TCA, the tricarboxylic acid cycle; O_2_, oxygen; GLUT1, Glucose transporter 1.

Tumor cells arise from transformations that fundamentally alter a cell’s proliferative and survival capacities. As such, the metabolic demands for cancer cells are different than those of a nonmalignant cell. In 1956, Warburg observed increased rates of glycolysis and lactate production in tumors, indicative of a preference for aerobic glycolysis over the more efficient oxidative phosphorylation pathway for generating ATP (10), a term later coined “The Warburg Effect”. Although less efficient than oxidative phosphorylation at ATP generation, the increased flux through aerobic glycolysis also generates metabolic intermediates necessary for supporting rapid proliferation, including glycerol and citrate for lipid biogenesis (11). The increased proliferative demands of tumor cells also increases rates of glutaminolysis to sustain anaplerosis, and this results in tumor metabolism of glutamine being higher than that of any other nonessential amino acid (12). The high utilization of these nutrients by tumor cells leads to competition for these resources with other cells, resulting in a glutamine restricted (13), glucose-poor, hypoxic, and acidic milieu (14, 15) which have been shown to suppress T cell infiltration and function (16–18), **Figure 3**. In addition, other immune subsets in the TME which are crucial to tumor-specific T cell activation, such as Dendritic Cells (DCs), are also adversely affected by the altered metabolic environment of the tumor (19, 20). Although the mechanism of immune cell suppression in the tumor is much more nuanced than simple nutrient depletion and competition, the unique milieu of the tumor requires that immune cells reshape metabolic flux to survive, which can then drastically affect their function (21). Targeting these nutrient pathways, summarized in **Figure 3**, may serve a dual purpose of not only reducing tumor growth, but also supporting the survival and antitumor efficacy of TCR-T.

In addition to metabolite synthesis, cells normally integrate information regarding nutrient availability and stress to regulate survival and proliferation; unfortunately, tumor cells have co-opted many aspects of these signaling cascades, including ones involving Mammalian Target of Rapamycin (mTOR), in order to sustain rapid division (22). mTOR has long been thought of as a master regulator of cell growth and longevity in response to nutrients and growth factors, and exists in two functionally distinct complexes, mTOR Complex 1 (mTORC1) and mTOR Complex 2 (mTORC2) (23), each with differing drug sensitivities (24, 25). Aberrant expression of mTOR and associated pathway components are very common in solid tumors (26–28), and contributes to tumorigenesis and growth (29). In addition, mTOR also plays a role in T cell differentiation and function (30), making it an attractive target for enhancing antitumor immunity. Strategies for modulating mTOR signaling also can serve to enhance fitness and cytotoxicity of TCR-T while concomitantly reducing tumor cell growth (31).

### Metabolic reshaping of the TME to enhance T cell fitness and function

The high level of glucose consumption by tumor cells provides not only fuel for glycolysis, but also increases flux through the Pentose Phosphate Pathway (PPP), which is consequently also elevated in malignant cells compared to normal cells (32). This pathway is crucial for generating intermediates for nucleic acid synthesis, nicotinamide-adenine dinucleotide phosphate (NADPH) for fatty acid synthesis (33), and generating glutathione to scavenge reactive oxidative species (ROS) (34), all of which are indispensable for rapidly dividing cells. Glucose-6-Phosphate Dehydrogenase (G6PD) is the rate limiting enzyme of the PPP, and its inhibition can induce autophagy (35) and senescence (36) in tumor cells, as well as inhibit their proliferation and metastasis (37). Interestingly, although T cells also proliferate rapidly upon activation, blockade of G6PD appears to generate superior CD8+ effector T cells which mediated a stronger tumor antigen-specific response, as well as increased proinflammatory cytokine secretion. In these T cells, the increased mitochondrial ROS is balanced by a concomitant increase in additional antioxidant enzymes to prevent oxidative damage (38). Taken together, these studies suggest that targeting the PPP through inhibition of G6PD can represent a “best of both worlds” situation in which tumor cells may not be as metabolically plastic as T cells due to their overreliance on specific metabolites, allowing T cells to leverage the shift in metabolic fuel to their benefit.

Evidence of plasticity in T cell metabolism has been demonstrated in the context of multiple metabolic pathways. Glutamine provides the fuel needed for the tricarboxylic acid (TCA) cycle, which in turn generates the intermediates needed for lipid, protein, and nucleic acid synthesis crucial for rapidly proliferating cells (39). Predictably, tumor cells rely heavily on this pathway for their survival, and indeed many tumors show evidence of being “addicted” to glutamine (40, 41). Treatment of tumor-bearing mice with glutamine antagonist JHU083, which impairs enzymes that require glutamine, induced not only durable antitumor effects, but also disrupted Warburg physiology by impairing glucose metabolism. Subsequently, glucose and glutamine concentrations both rose within the tumor, followed by a parallel decrease in hypoxia. T cells treated with glutamine antagonist showed increased markers of activation and memory, and demonstrated enhanced cytokine production upon restimulation. Interestingly, the T cells were found to be able to switch to utilizing acetate as a carbon source for the TCA cycle, a feat which the tumor cells were not able to replicate (42). In another study, the pharmacologic inhibition of glutamine transporter was able to impair tumor growth and also increase the concentration of glutamine in the tumor. However, as T cells adapted by upregulating a separate amino acid transporter, they were able to continue uptake of glutamine and were not affected by the drug – in addition to demonstrating enhanced activation and cytotoxic functions (43). Thus, although T cells and tumor cells rely on increased flux through many of the same pathways for survival and function, the superior metabolic plasticity of T cells can allow for metabolic interventions which reduce tumor fitness while enhancing T cell effector capabilities.

As part of Warburg physiology, large amounts of lactic acid are generated as byproducts of aerobic glycolysis, which is subsequently secreted from the tumor cells and contributes to the acidification of the tumor compartment (44). This increased concentration of lactic acid impairs cytotoxic function of both T cells and natural killer (NK) cells (45) by impairing their ability to secrete the lactic acid byproduct of their own increased glycolytic flux following activation, the secretion of which requires a concentration gradient (46). Treatment with Diclofenac, which inhibits the lactic acid transporters Monocarboxylate Transporter 1 and 4 (MCT1 and MCT4, respectively), enhanced the efficacy of checkpoint blockade immunotherapy through improved tumor control and cytokine secretion. In addition, T cells treated with diclofenac shifted their glucose flux to the TCA cycle and increased oxygen consumption, allowing them to retain their cytotoxic functions. However, it should be noted that proliferation of T cells was reduced following treatment with diclofenac (47), which could indicate that the timing of administration could be crucial in enhancing the efficacy of lactic-acid targeting therapies. Another paper also corroborated these findings through RNA interfering (RNAi)-nanoparticle mediated knockdown of lactate dehydrogenase (LDHA) in tumor cells, which also neutralized tumor pH and increased the infiltration and cytotoxicity of NK and T cells. Interestingly, the authors also conducted these experiments in immunocompromised mice and showed no effect on tumor growth, demonstrating that cytotoxic immune infiltrate was necessary for potentiating the antitumor effect of targeting lactic acid metabolism (48). Although targeting lactic acid metabolism may negatively affect proliferation of endogenous T cells, TCR-T can be administered after lactic acid transporter inhibition “preconditioning”, which could potentially render the tumor milieu more amenable to the survival and efficacy of the transferred cells.

In addition to the metabolites mentioned previously, the anabolism/catabolism of other nutrients and amino acids can also drive immunosuppression in the TME and thus serve as potential targets for metabolic therapy. One example is the role of Indoleamine 2,3-dioxygenase (IDO), the first and rate-limiting step of tryptophan degradation as part of the kynurenine pathway, which fuels many essential biological processes including energy metabolism and generation of the co-factor Nicotinamide adenine dinucleotide (NAD+) (49). IDO is elevated in many tumor types and is correlated with poorer prognoses in solid tumors (50), and a higher kynurenine-to-tryptophan ratio is associated with shorter survival in patients with leukemia (51). One possible explanation for this association is that the tryptophan/kynurenine axis has been associated with multiple immunoregulatory effects – kynurenine can bind to the aryl hydrocarbon receptor (AHR), which skews T cells towards a immunosuppressive regulatory T cell (Treg) phenotype (52); additionally, it can induce exhaustion and immune checkpoint expression in T cells (53) and drive conversion of dendritic cells (DCs) towards a tolerogenic phenotype (54). In T cells specifically, IDO-mediated tryptophan deprivation was found to induce autophagy and inhibit both mTOR and protein kinase C theta (PKC-Θ) (55), which are crucial for T cell activation and proliferation (56). IDO inhibition in combination with a tumor vaccine was found to be able to convert suppressive Tregs into Th17-like cells in tumor draining lymph nodes of tumor-bearing mice, and this treatment both enhanced CD8 T cell activation and reduced tumor growth (57). Given their preclinical promise, IDO inhibitors which target IDO1 or the isoenzyme IDO2 have been and are currently being investigated in clinical trials. The ECHO-301/KEYNOTE-252 trial found that treatment with the IDO1 inhibitor Epacadostat did not improve overall survival or progression free survival in melanoma (58), which brings into question the utility of IDO inhibitors in a clinical setting. These disappointing results could be explained by several factors: the inability of Epacadostat to completely inhibit IDO1 even at the highest dose, the compensatory expression of other tryptophan-degrading enzymes such as IDO2, and patient cohort selection (59). Subsequent studies have focused on targeting other metabolites in the kynurenine pathway, including kynurenine itself. Treatment of tumors *in vivo* with an enhanced kynureninase to degrade kynurenine increased infiltration and proliferation of cytotoxic CD8s, resulting in improved tumor control in a variety of tumor types. Interestingly, the treatment had no effect on tumor growth in immunocompromised mice, suggesting that the antitumor mechanism of kynureninase is contingent upon the presence of immune cells (60). The example of IDO inhibitors demonstrates the importance of understanding compensatory mechanisms and possibility of targeting alternative components of the same pathway when investigating the effects of metabolic perturbations.

### Increasing neoantigen presentation in the TME

Effective antitumor immune therapy is predicated on the function of dendritic cells (DCs), which uptake and present tumor antigen to activate naive T cells and induce their proliferation (61). DC ablation drastically reduces the activation of memory T cells - whose reactivation threshold is much lower and postulated to be DC-independent - in response to infections (62), thus suggesting that DCs play a role in maximizing the efficacy of T cell responses. In TCR-T, endogenous DCs can present tumor neoantigens to mobilize new cohorts of T cells of diverse TCR clonotypes, which is crucial to address epitope spread – especially since loss of target antigen is a common tumor mechanism of resistance in patients who relapse post-ACT (63). In the tumor, certain yet unknown tumor factors induce upregulation of scavenger receptors on DCs, increasing their uptake of lipoproteins and increasing their internal lipid content. These lipid-loaded DCs were shown to be unable to process and present antigen effectively, and were drastically impaired in their ability to activate T cells. Targeting fatty acid synthesis by impairing acetyl-CoA carboxylase, and thus forcing DCs to utilize exogenous lipids for triglyceride synthesis, reversed this effect (64). However, since proliferating T cells also increase their requirements for fatty acid synthesis to sustain division and inhibiting this pathway has been shown to be detrimental to cell expansion (65), systemic inhibition of acetyl-CoA carboxylase would have to be carefully timed to avoid interference with TCR-T.

A similar tale of functional balance can be told for the role of adenosine monophosphate-activated protein Kinase (AMPK) pathway in DC activation and T cell effector capabilities. AMPK is crucial to regulating energy balance within the cell, turning on ATP-producing catabolic pathways when cellular stress reduces the amount of ATP available (66). AMPK activation is crucial for protein kinase B (PKB; also known as AKT) activation in response to cellular stress (67), which in turn phosphorylates and inactivates Glycogen synthase kinase-3 beta (GSK3β), a negative regulator of cAMP response element-binding protein (CREB). This results in CREB-mediated enhancement of anti-inflammatory IL-10 transcription while reducing proinflammatory cytokine transcripts through reduction of Nuclear factor kappa-light-chain-enhancer of activated B cells (NF-κB) signaling (68). Phosphorylation of the alpha subunit of AMPK (AMPKα) is required for AMPK activation (69). Knockdown of isoform 1 of this subunit, AMPKα1, in macrophages and DCs enhanced antigen presentation, decreased the production of the anti-inflammatory cytokine interleukin 10 (IL-10), and increased production of IL-17 as well as interferon gamma (IFN-y). This favored the skewing of activated helper T cells to T helper 1 (Th1) and 17 (Th17) phenotypes (70). In a tumor setting, these AMPKα1 deficient macrophages and dendritic cells would be poised to increase tumor antigen presentation, and induce a favorable proinflammatory phenotype of T cells. However, the effects of decreased AMPK in T cells remain less clear, but is necessary to consider if treatments targeting AMPK are to be administered concurrently with TCR-T. Although AMPKα1 deficient CD8 cells produced more proinflammatory IFN-y and IL-17A, they also had impaired viability compared to control cells when subjected to metabolic stress (71). This finding was corroborated by another study, in which the authors demonstrated that this requirement for AMPKα1 to drive T cell functionality was dependent on glucose concentration – that is, T cell impairment was observed in AMPKα1 deficient cells when glucose was scarce (72), as is the case in the tumor microenvironment. Interestingly, metformin, an AMPK agonist, has been shown to directly enhance antitumor function in tumor antigen specific T cells, increasing polyfunctionality and protecting T cells from apoptosis and exhaustion. The same effect was not noted in immunodeficient mice, suggesting that this antitumor effect was driven specifically by the presence of immune cells (73). Taken together, these studies indicate that the balance of AMPK signaling must be modulated very carefully; while increased signaling may lead to better immune cell survival and reduce flux through glycolytic pathway in cancer cells (74), it can also contribute to reducing the effects of cell stress signaling which can lead to tumor cell survival. Future treatment regimens may consider pretreating the T cells in TCR-T with an AMPK agonist to increase stress resistance, while utilizing tumor metabolomics to consider if AMPK is a viable target in each individual tumor setting.

## Strategies for improving functionality and persistence of adoptively transferred T cells

In TCR-T, human T cells are engineered with a TCR of interest and then infused into the patient, where the hope is that those cells will traffic to the tumor and surrounding lymph nodes, setting up long-lived cytotoxic populations which will attack the tumor. In order for this therapy to be successful, the T cells must be able to survive in the TME, and thus are subject to many of the same pitfalls that plague immunotherapy in general – namely, exhaustion of effector cells, poor viability, and loss of effector function (75). The competition for glucose with tumor cells in the TME results in T cells with reduced capacity to produce IFN-y and decreased signaling through glycolysis and nutrient sensing pathways (76). Engagement of inhibitory immune checkpoint molecules such as Cytotoxic T-Lymphocyte Associated Protein 4 (CTLA-4) and Programmed Cell Death Protein 1 (PD-1) on T cells inhibits the glycolysis and amino acid metabolism which is crucial to effector differentiation and function (77). Hypoxia in the TME can upregulate the ligand for PD-1, Programmed death-ligand 1 (PD-L1) on tumors (78–81), as can glucose-depleted conditions (82), which engage inhibitory checkpoints on T cells and lead to their “exhaustion”. Even though the TME is known to be immunosuppressive and poor in nutrients, the survival and persistence of infiltrating immune cells is crucial to the sustained effectiveness of TCR-T. Already, blockade of CTLA-4, PD-1, and PD-L1 are being utilized to reinvigorate the dysfunction seen in exhausted T cells, and can restore effector T cell metabolic profiles (77) while dampening glycolytic flux in tumor cells (76). Tumor infiltration of T cells can also be influenced by nutrient changes in the TME, including hypoxia-mediated lactate accumulation which inhibits T cell migration (83), and aberrant lipid metabolism which negatively impacts function and migration of a variety of immune subsets (84). Here, we will summarize some strategies to “armor” T cells against metabolic challenges posed by the TME.

### Targeting glucose metabolism to increase T cell functionality

**Figure 4:**
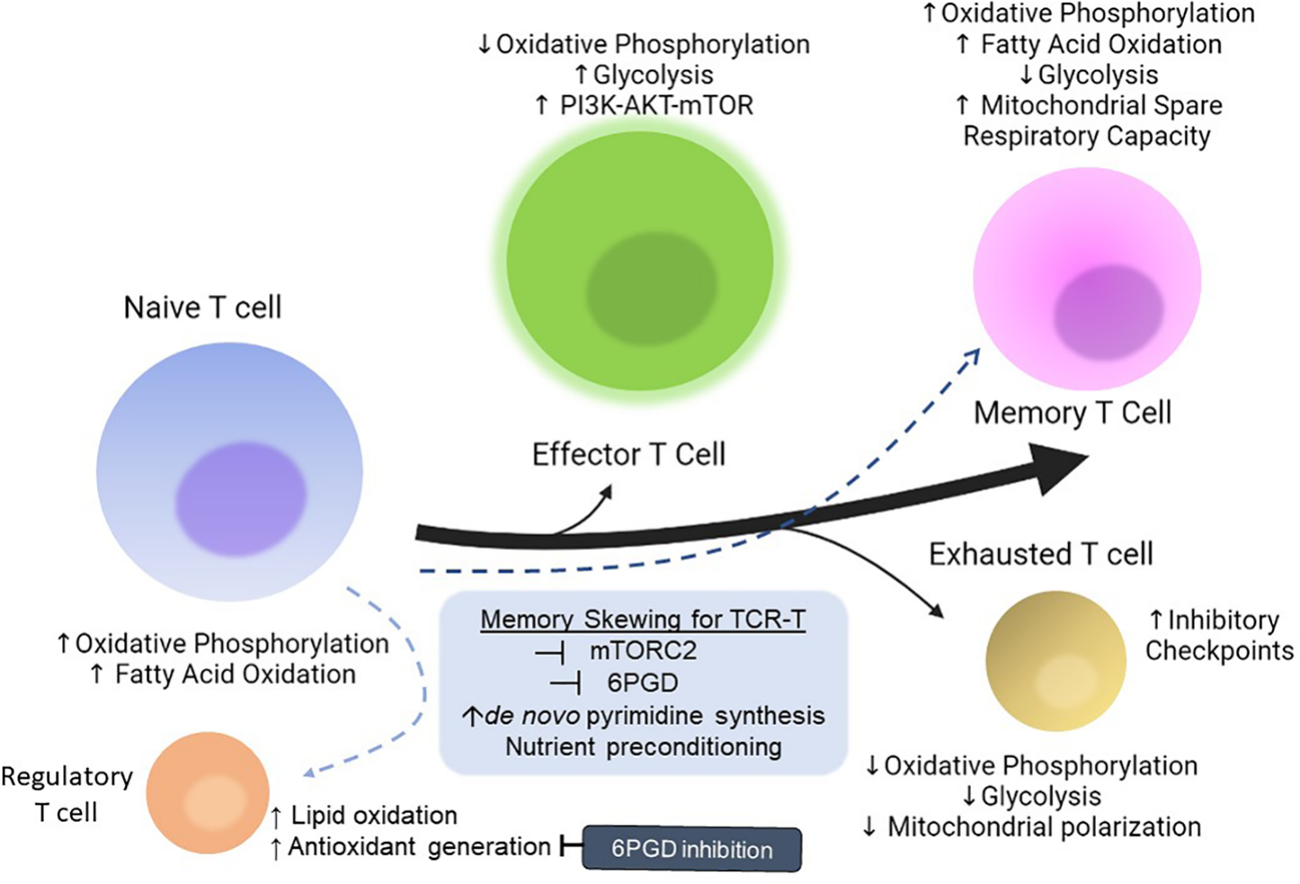
Metabolic phenotypes of T cells and strategies for skewing towards memory phenotype. Depiction of changes in T cell metabolism upon activation, and highlighting differences in metabolism upon transition either to a memory phenotype or an exhausted effector cell. Strategies for improving memory phenotype skewing are also depicted in the blue box. 6PGD, 6-phosphogluconate dehydrogenase; PI3K, phosphoinositide 3 kinase; AKT, protein kinase B; mTOR, mammalian target of rapamycin; mTORC2, mTOR Complex 2.

Upon activation, T cells shift their metabolism from oxidative phosphorylation (OXPHOS) to glycolysis to meet energy requirements (85, 86), **Figure 4**. After the effector phase is completed, T cells yet again shift to rely on fatty acid metabolism for energy when they differentiate into memory T cells (T_mem_) (87). Inhibition of glycolysis during antigen encounter drives T cells towards a memory phenotype (88) (**Figure 4**), but also reduces effector function (89). Glucose metabolites are also utilized in the PPP, which provides crucial intermediates for cell survival and growth, and scavenges ROS produced during cell proliferation. Inhibition of 6-phosphogluconate dehydrogenase (6PGD), the enzyme catalyzing the second step of the PPP, CD8+ T cells increased mitochondrial content and mitochondrial ROS production balanced by increased antioxidant production to protect against oxidative damage. Functionally, these cells increased IFN-y production and granzyme B, and showed superior effects against both tumors and infections, consistent with a T Effector Memory (T_EM_) phenotype. The cells also increased their expression of glucose-transporter 1 (GLUT1) to increase glucose uptake (38), which is crucial in a TME where there is tremendous competition for this crucial nutrient (76) (**Figure 3**). Blockade of 6PGD may also have the benefit of reprogramming immunosuppressive Tregs - which rely heavily on the antioxidant capacities of the PPP to balance the ROS produced by their fuel of choice – lipid oxidation - into Th1, 2, and 17 cells, which improved antitumor responses (90) (**Figure 4**).

### Preconditioning regimens to increase T cell survival in the TME

In previous sections, we have explored the evidence for T cell metabolic plasticity which allows them to adjust flux through various pathways to adapt to environmental cues. This plasticity can not only be leveraged to favor survival of T cells over tumor cells, but also to induce metabolic changes which prepare T cells for the nutrient challenges they will face in the TME. Although glucose has been shown to be essential for T cell effector function, transient glucose restriction (TGR) in activated cells can help enhance antitumor function. In a mouse model of lymphoma, adoptively transferred effector CD8s preconditioned in low glucose conditions were able to mediate complete clearance of tumor, with an enhanced effector phenotype and increased number in circulation. TGR T cells redirected their utilization of glucose carbons to the TCA cycle rather than glycolysis, resulting in a drastic increase in ATP generation, and were better poised to handle oxidative stress due to the sustained synthesis of the reactive oxidative species (ROS) scavenger glutathione – two characteristics which allow these T cells to better survive in the TME compared to cells which have not undergone preconditioning. Metabolomic interrogation of these TGR effector cells showed that these cells were able to enhance their glucose uptake following re-exposure to glucose and increase flux through the PPP (91), both of which can boost effector function and improve ability to compete for nutrients.

In a similar vein, glutamine restriction can also yield comparable results to glucose restriction. T cells cultured in glutamine depleted conditions or treated with inhibitors of glutamine metabolism were shown to increase survival and promote tumor clearance. Phenotyping of the tumor infiltrate showed that glutamine restricted T cells displayed lower levels of exhaustion markers and increased proliferation, cytokine production, and markers associated with long-lived memory populations. Metabolically, these cells showed increased mitochondrial spare respiratory capacity, reduced mitochondrial ROS, and increased glycolytic flux, indicating that glutamine restriction can skew cells metabolically towards a memory-like phenotype with greater self-renewal and antitumor capabilities (92). Just as in the case of glucose restriction, glutamine restriction can rewire T cells to utilize available nutrients more effectively while also increasing oxidative stress tolerance, giving preconditioned effector cells an edge over their non-pretreated counterparts in the TME.

During glycolysis, the generation of pyruvate from phosphoenolpyruvate (PEP) by pyruvate kinase generates one molecule of ATP (93). Knockdown or pharmacological inhibition of PEP-generating enzymes T cells led to a decrease in calcium flux upon activation in T cells, which showed diminished cytokine production and effector capacities. In T cells, overexpression of phosphoenolpyruvate carboxykinase 1 (PCK1), which can generate PEP from the TCA cycle intermediate oxaloacetate, improved function in both CD4s and CD8s both in terms of measured calcium flux and antitumor effects. Interestingly, overexpression of PCK1 only increased intracellular PEP in low glucose conditions (94), making it a promising modification for increasing efficacy in the glucose-depleted TME while preventing aberrant metabolism in the periphery. This suggests that despite low levels of crucial nutrients in the TME, strategies for increasing expression of intermediates in key pathways can obviate some of the effects of nutrient deprivation in T cells.

Cell therapies comprising greater populations of memory cells compared to terminally differentiated effectors can yield greater antitumor efficacy and long-term durability (95). Other studies have found that a naïve/stem cell memory population resulted in reduced cytokine release syndrome and improved antitumor efficacy compared to bulk unselected T cell infusion, which comprises mostly terminally differentiated effectors; however, stem cell memory T cells are extremely rare in circulation and their expansion requires additional engineering steps; additionally, the initial cytotoxic functions of these cells are not as robust as bulk effectors (96). Both effector/central memory cells and naïve/stem cell memory populations exhibit the ability to expand and differentiate, making them very valuable for cell therapy. One of the hallmarks of a memory T cell is their ability to respond rapidly and robustly to antigen rechallenge. Critical to this rapid recall response is carbamoyl-phosphate synthetase 2, aspartate transcarbamylase, and dihydroorotase (CAD), the rate-limiting enzyme of the *de novo* pyrimidine synthesis pathway. Inhibition of CAD decreases available pre-rRNA, which limits the ribosomal biogenesis required for synthesizing new proteins to support proliferation and cytokine production. Overexpression of CAD was shown to enhance proliferation and improve cytokine production upon rechallenge (97). This suggests that methods of increasing flux through the *de novo* pyrimidine synthesis pathway *ex vivo* could yield an improved cohort of memory cells for infusion (**Figure 4**), with enhanced antitumor and proliferative capacities, potentially ameliorating the need for repeated infusions.

Alternate pathways through which T cells generate fuel in glucose-depleted conditions can also identify specific targets for preconditioning regimens to further enhance T cell function. T cells can utilize acetate as an alternative to glucose in glucose-poor environments, and *ex vivo* exposure to acetate can increase histone acetylation and chromatin accessibility to epigenetically remodel exhausted T cells into active effectors again. These cells show increased cytokine production and promote tumor clearance (98). Inosine can also be utilized by T cells as an alternative to glucose, and treatment with inosine after adoptive transfer also enhances antitumor effector function in tumor models that are unable to metabolize inosine (99). Although it could be speculated that inosine preconditioning would also enhance T cells’ ability to utilize this alternative fuel source in a tumor setting, studies still need to be completed to test this hypothesis. Additionally, the ability of some tumors to also metabolize inosine (100, 101) and thus compete with T cells for this fuel source would also limit its utility.

### Strategies for navigating the complexities of mTOR signaling to increase T cell fitness

**Figure 5:**
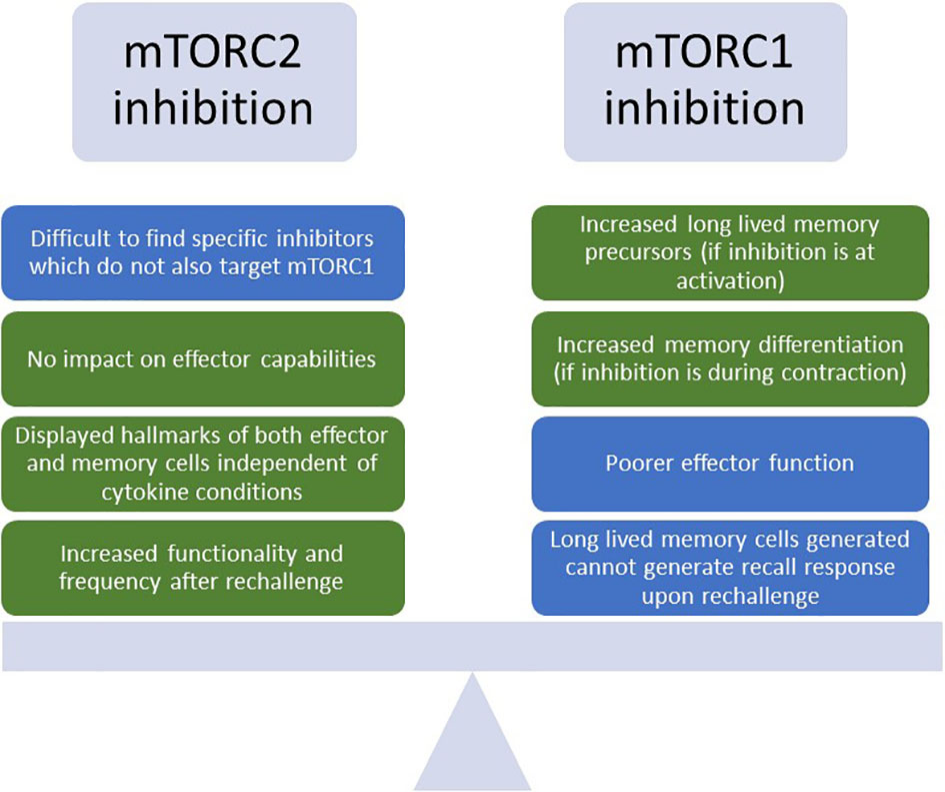
Benefits and drawbacks of inhibiting mTORC1 or mTORC2 to enhance T cell phenotypes for TCR-T. Depiction of the challenges and advantages of inhibiting either mTORC1 (mTOR Complex 1) or mTORC2 (mTOR Complex 2) for T cell enhancement. Green boxes indicate benefits, and blue boxes indicate challenges. mTOR, mammalian target of rapamycin.

During T cell activation and differentiation, mTOR signaling through mTORC1 and mTORC2 have differing effects on T cell fate and phenotype. Constitutive mTORC1 activation in T cells generates highly potent effector CD8s, but at the cost of inhibiting transition to a memory phenotype (102, 103). However, although inhibition of mTORC1 led to generation of long-lived memory cells which exhibited poorer effector function, these cells also failed to display a hallmark of memory cells – ability to generate a recall response upon rechallenge. Interestingly, in the T cells where constitutive mTORC1 signaling decreased memory formation, administration of rapamycin – an mTORC1 inhibitor - was able to re-enable the formation of memory recall responses (102). A similar study also showed that rapamycin treatment during initial activation and expansion favored the generation of memory precursors, while treatment during the contraction phase favored the differentiation of memory cells (104). The importance of precisely timing this inhibition is further supported by the observation that mTORC1 signaling is inexorably linked to glucose uptake and glycolysis, and treatment with rapamycin early upon activation both impairs glucose metabolism and decreases perforin production (105). These studies suggest that the inhibition of mTORC1 can be leveraged to achieve both a potent effector response and generation of long-lived memory through the carefully timed targeting of mTORC1 (**Figure 5**).

Due to the complexity of targeting mTORC1 to enhance potent and long-lived T cell responses, mTORC2 has also emerged as a potential target. T-cell specific knockdown of Rapamycin-insensitive companion of mTOR (Rictor), a binding partner of mTORC2 crucial to its activation, was able to generate robust T cell responses similar to wild type controls upon vaccination, indicating that deficiencies in mTORC2 did not affect effector capabilities. Interestingly, upon rechallenge, these cells displayed increased markers of memory, robust cytokine production, and were present at a higher frequency in the spleen. This is possibly due to the fact that mTORC2 prevents activation and nuclear translocation of Forkhead Box O1 (FOXO1), a transcription factor which is responsible for upregulation of memory-associated transcripts like L-selectin (CD62L) and C-C Motif Chemokine Receptor 7 (CCR7) (106). Additionally, they displayed hallmarks of both effector and memory T cells, with increased glycolytic flux and enhanced spare respiratory capacity (SRC) for long-term survival, regardless of whether the culture media contained cytokines favoring effector or memory formation (102). This study suggests that inhibition of mTORC2 can lead to the generation of T cells bearing favorable characteristics of both potent effectors and long-lived memory, enhancing their survival in the TME while also decreasing their dependence on exogenous cytokines for maintenance of phenotype. Thus, targeting of mTORC2 could be a viable alternative to mTORC1 inhibition in generating cells endowed with both intact effector functions and memory-like persistence, with greater metabolic resilience to the challenges posed by the TME; however, a challenge remains in identifying specific mTORC2 inhibitors which do not target mTORC1 as well (107) (**Figure 5**).

### Personalized strategies for assessing T cell metabolic vulnerabilities

In recent years, advances in sequencing and analysis technologies have made it possible to conduct large-scale screens at a reduced cost, bringing truly personalized medicine within reach. As mentioned previously, different tumors will have varying metabolic vulnerabilities, and T cells from individual patients will also display similar diversity in sensitivities. As such, it may be prudent to leverage *in vitro* pharmacological or genetic screens to determine optimal treatment regimens for personalized metabolic medicine. A recent study described insights gained from such a screening process, where T-cell and tumor cells were placed in coculture in the presence of 240 different pharmacological perturbations to assess which compounds influenced T cell activation. Not only were they able to gain understandings about vulnerabilities of T cells and their underlying mechanisms, but they were also able to identify compounds in which T cells were differentially affected compared to tumor cells. For example, in the context of a B16-OVA tumor with OT-1 T cells, glutathione peroxidase 4 (GPX4) inhibition induced ferroptosis in CD8+ T cells while leaving tumor cells mostly unaffected, and this sensitivity was attributed to acyl-CoA synthetase long-chain family member 4 (ACSL4) (108), one of the key regulators of ferroptosis (109). One could imagine a scenario in which patient T cells could be initially screened against either tumor organoids or cell lines to identify metabolic enhancements which would benefit T cell survival over that of tumors, or to detect susceptibilities. Through performing personalized tumor-T cell screens, T cells for TCR-T can be engineered to reduce their metabolic vulnerabilities, and concurrent pharmacological interventions to target specific tumor vulnerabilities can be identified.

## Strategies for improving TCR-T screening and engineering

The fundamental principle behind TCR-T is that tumor-specific TCRs can be engineered onto a bulk population of T cells, and this population can then be redirected to target the tumor while sparing healthy tissue (110). However, this means that TCR discovery is also of high importance – TCRs must not only be specific for the tumor antigen, but also induce cytotoxicity and cytokine production in T cells once engaged. TCR signal strength has been correlated with T cell functional state, with high-strength interactions inducing inhibitory receptors and acquiring a molecular profile associated with dysfunction (111), suggesting that selecting TCRs with higher affinity may reduce efficacy of the final cell product. As such, methods for identifying TCRs with optimal affinity and influence on T cell phenotype can be very time consuming. These methods can include assessing individual TCR affinity, functional avidity, and conducting cytotoxicity assays to ensure that engineered TCRs have a high enough affinity to detect tumor targets, but not so elevated that it adversely affects their function (112). Understanding how metabolism can influence T cell sensitivity to antigen can decrease TCR screening time, and perhaps increase the number of TCRs that enter the final stages of preclinical evaluation to broaden the targetable patient population.

Another challenge in TCR-T has been optimizing the phenotype of the TCR-engineered T cell population throughout the expansion process to yield long-lived cells which will persist and carry out effector functions in their new niche. It has been found that a central memory phenotype of adoptively transferred T cells was optimal for obtaining the best antitumor responses in patients (113); however, a diverse starting population of T cells poses a challenge as to how to induce this profile for therapy. Previously, we have examined how metabolism plays a crucial role in T cell differentiation into memory and effector subsets, and this knowledge can also be applied to the engineering process to enhance efficacy of the final TCR-T product.

### Fine tuning peptide sensitivity of TCRs

T cell sensitivity to their cognate peptide/major histocompatibility molecule (MHC) appear to be governed by both intrinsic and induced factors – there is an intrinsic affinity of a TCR for its ligand, but also induced dynamic TCR clustering (114), both of which modulate the strength of activation. As such, a single T cell clone has the ability to differentiate into both high avidity and low avidity effectors, suggesting that the sensitivity of a T cell can be modulated at the time of activation and is not dependent on the TCR alone (115). After this period of plasticity, T cells develop an activation threshold “set point”, whereupon encounters with sub-threshold or supra-threshold levels of antigen result in apoptosis (116). This poses many issues for TCR therapy, from the initial screening needed for identifying optimal TCRs based on functional avidity, to the engineering process for clinical products. However, understanding the mechanism through which T cells are able to adjust their sensitivity can allow for enhanced cell therapy products and even new applications.

Upon activation, naïve T cells typically switch their metabolism from primarily oxidative phosphorylation to glycolysis (117). However, upon strong peptide stimulation, flux through both OXPHOS and glycolysis were increased. This increased OXPHOS upregulated the transcription factor NFATC1 leading to an increased production of IL-4, which signaled in an autocrine manner to reduce functional avidity of high-peptide-dose stimulated cells. IL-4 neutralizing antibodies were able to block this effect and restore sensitivity (118). Physiologically, the production of IL-4 and its autocrine signaling in cells that have encountered high peptide stimulation can serve as a mechanism for inducing tolerance in the periphery. This has implications for TCR-T, where TCR discovery efforts may be hindered by the activation “set point” of cells used to screen for reactivity. Of course, recent advancements in screening capabilities using phage display or yeast libraries remove the need for using T cells or APCs for initial TCR screening, which allows for identification of putative antigen-reactive TCRs without being affected by T cell or APC phenotypes; however, these systems may not be optimal for low-affinity interactions (119), and T cell-based systems are still needed to validate TCRs for functional relevance. In addition to the complexities of TCR discovery, when it comes to manufacturing the cell product, the activation threshold of different T cells in the starting population may be different even when bearing the same TCR, affecting the sensitivity of the final product (118). It could be postulated that monitoring and adjusting IL-4 levels could help alleviate some of these concerns. Additionally, the fine-tuning of activation threshold can be seen as a blessing, in which T cells could theoretically be able to discern between increased levels of antigen on tumor cells versus low physiological levels of antigen on normal tissue. Due to the challenges of identifying TCRs against antigens specific to the tumor, being able to modulate sensitivity of T cells to target overexpressed tumor antigens such as HER-2 (120) would greatly expand TCR-T treatment to more indications.

### Optimizing expansion culture conditions to improve T cell functionality

Cell manufacturing for TCR-T incorporates large-scale cell culture capable of expanding TCR-engineered T cells in preparation for clinical infusion. Multiple factors other than the TCR itself have been shown to influence the final cell product, and composition of media in which cells are expanded can play a large role in their antitumor capabilities (121). It has been shown that expanded T cells *in vitro* have a different metabolic profile than cells activated in physiologic conditions, with the former acquiring more of a Warburg-like metabolism and the latter displaying higher rates of oxidative metabolism (122). The differentially active metabolic and signaling pathways between cultured and physiologically conditioned cells could raise concerns regarding the fitness of these cultured cells once placed into a physiologic setting, such as patient infusion. A recent study exploring the impact of several different media formulations on cell therapy products found that exposure to ascites from ovarian cancer patients reduced IFN-y and tumor necrosis factor alpha (TNF-a) production by T cells regardless of expansion condition. However, there were differences in the extent to which ascites exposure reduced viability, cytokine production, and mitochondrial activity, suggesting that different media formulations with varying levels of nutrients can affect T cell resilience in TME-like conditions (123). Additionally, supplementing with interleukin-7 and -15 in culture during T cell expansion can promote a central memory phenotype and robust antitumor function (124); IL-7 has been found to induce expression of glycerol channel aquaporin 9 (AQP9) on memory T cells, which imports glycerol for fatty acid esterification and triglyceride synthesis required for survival (125). Additional studies have shown the synergistic potential of IL-21 with IL-15 in enhancing cell function and antitumor effects (126, 127); T cells cultured in IL-21 media increased fatty acid oxidation over glycolysis, skewed towards a central memory phenotype, and displayed reduced exhaustion markers (128). Thus, cytokine supplementation in media can have profound effects on T cell metabolism and phenotype, and can be utilized to generate cell populations with enhanced self-renewal and differentiation capabilities. To date, there is no standardized expansion media condition for cell therapy production across the field. Optimizing expansion conditions by adjusting the levels of key metabolites can generate cell products which persist better in the TME and retain more of their antitumor function under immunosuppressive conditions.

## Discussion

Harnessing the immune system for attacking tumors is an area of active investigation, and TCR-T is a promising treatment modality which endow T cells with new specificity against tumor antigens. This has the potential to treat even advanced disease; indeed, a recently published case report highlighted the ability of Kirsten rat sarcoma virus (Kras) G12D mutation-targeting TCR-T to mediate regression of metastatic pancreatic cancer, with the response ongoing 6 months post-treatment (3). To ensure more cases of tumor regression like this one, there needs to be a deeper understanding of how infused T cells interact with the immunosuppressive TME, which has an altered nutrient and metabolite profile. Metabolism plays a key role in T cell fitness and effector capacity, and certain alterations in nutrient conditions can abrogate activation altogether. However, these metabolites also play a role in tumor cell survival, and finding the precise metabolic targets which enhance T cell function at the expense of the tumor can lead to new treatments given alongside TCR-T to improve efficacy. In this review, we highlight several methods of targeting both tumor and T cell metabolism to improve antitumor response. However, one of the largest challenges in the immune-oncology sphere is tumor heterogeneity, and there is no guarantee that metabolic vulnerabilities will be consistent even within the same tumor, let alone across indications. Personalized screening can help to identify unique susceptibilities specific to tumors and patient T cells; however, it will need to keep in mind the limitations set by tumor evolution and heterogeneity. In addition, metabolism is a matter of balance, and as seen with the example of mTOR targeting, perturbations must be timed precisely to be able to yield the desired phenotype. While the plasticity of T cells allows them to acclimate to different nutrient conditions, metabolic interventions during a T cell response raises the possibility of skewing towards immunosuppressive populations such as Tregs, as is the case with mTORC1 inhibition in certain conditions (129). New methods of three dimensional automated coculture screening can more closely mimic dynamics of T cell activation and help identify the effects of individual metabolic perturbations on T cell function and phenotype and identify potential targets for enhancement (130), and can be applied for personalized medicine as well. Further studies into the temporal relationship between metabolism, trajectory of activation, and *in vivo* phenotype will shed further light on potential treatment regimens. Due to the nature of T cell engineering and expansion for cell therapy, there is a unique window of opportunity to modulate the phenotypic profile of T cells prior to infusion, in addition to post-infusion systemic treatment. Additionally, most metabolic enhancements, such as nutrient preconditioning, are very cost-effective and simple to implement – without the need for lengthy regulatory approvals that accompany genetic engineering.

Future TCR-T approaches will seek to shorten engineering time, improve cell function and phenotype, and broaden the applicability of therapy to more patients. Although initial trials with TCR-T utilized a patient’s own cells for engineering (autologous cell therapy), this requires that engineering and expansion be completed for each individual, which is resource and time intensive. There has recently been a push towards “off the shelf” allogeneic products, whereby healthy donor cells can be engineered to form a bank of cells which can then be infused into any HLA-matched patient, reducing time and engineering costs; additionally, the availability of large numbers of these cells allows for repeat dosing (131, 132). However, as with any transplant scenario, there is concern for graft-versus-host disease (GVHD), and much effort has been put into preventing transferred cells from attacking healthy host tissue due to alloreactivity. In addition to current engineering controls to test TCRs for alloreactivity (133, 134) and suppress endogenous TCR expression (135, 136), infusing cells with a memory phenotype, which is favored for cell therapy, can reduce the chances of GVHD occurrence (137–139). Above, we have discussed multiple methods through which to influence T cell metabolism to generate memory cells which are more resilient and polyfunctional, many of which can be easily translated into current engineering protocols. Metabolic interventions represent a promising treatment modality to augment the efficacy of TCR-T, leading to a more robust cell product that is more resilient to the immunosuppressive effects of the TME and demonstrate superior antitumor efficacy.

## Author contributions

WM, conceptualization, writing‐original draft, and figure design.

## Acknowledgments

The author would like to acknowledge the research team at BioNTech for their support of this work.

## Conflict of interest

The author is an employee of BioNTech USA.

## Publisher’s note

All claims expressed in this article are solely those of the authors and do not necessarily represent those of their affiliated organizations, or those of the publisher, the editors and the reviewers. Any product that may be evaluated in this article, or claim that may be made by its manufacturer, is not guaranteed or endorsed by the publisher.
